# Disparate metabolic effects of blackcurrant seed oil in rats fed a basal and obesogenic diet

**DOI:** 10.1007/s00394-014-0775-z

**Published:** 2014-10-14

**Authors:** Adam Jurgoński, Bartosz Fotschki, Jerzy Juśkiewicz

**Affiliations:** Division of Food Science, Institute of Animal Reproduction and Food Research, Polish Academy of Sciences, 10 Tuwima Street, 10-748 Olsztyn, Poland

**Keywords:** Blackcurrant seed oil, Essential fatty acids, Lipid metabolism, Liver function, Caecum, Rats

## Abstract

**Purpose:**

It was hypothesised that blackcurrant seed oil beneficially modulates metabolic disorders related to obesity and its complications. The study also aimed to investigate the potentially adverse effects of an unbalanced diet on the distal intestine.

**Methods:**

Male Wistar rats were randomly assigned to four groups of eight animals each and were fed a basal or obesogenic (high in fat and low in fibre) diet that contained either rapeseed oil (Canola) or blackcurrant seed oil. A two-way analysis of variance was then applied to assess the effects of diet and oil and the interaction between them.

**Results:**

After 8 weeks, the obesogenic dietary regimen increased the body weight, altered the plasma lipid profile and increased the liver fat content and the plasma transaminase activities. In addition, the obesogenic diet decreased bacterial glycolytic activity and short-chain fatty acid formation in the distal intestine. Dietary blackcurrant seed oil improved the lipid metabolism by lowering liver fat accumulation and the plasma triglyceride concentration and atherogenicity as well by increasing the plasma HDL-cholesterol concentration. However, in rats fed an obesogenic diet containing blackcurrant seed oil, the plasma HDL-cholesterol concentration was comparable with both rapeseed oil-containing diets, and a significant elevation of the plasma transaminase activities was noted instead.

**Conclusions:**

The obesogenic dietary regimen causes a number of metabolic disorders, including alterations in the hindgut microbial metabolism. Dietary blackcurrant seed oil ameliorates the lipid metabolism; however, the beneficial effect is restricted when it is provided together with the obesogenic diet, and a risk of liver injury may occur.

## Introduction

Obesity is one of the most challenging health problems worldwide which leads to the development of many different medical conditions, like cardiovascular disease (CVD) and the metabolic syndrome, on the one hand, and gastrointestinal disorders including colorectal cancer, on the other hand. A well-established risk factor associated with all the aforementioned diseases is an energy-dense dietary pattern rich in fat and refined carbohydrates and low in fibre [1, 2]. Due to its high energy density, fat has been recognised as an important macronutrient whose dietary amount and type can be easily modified. It is generally accepted that an overabundance of saturated fatty acids (SFAs) can be harmful for the body, whereas polyunsaturated fatty acids (PUFAs) are considered to be rather beneficial [1, 3]. Although the exact energy yield of the individual fatty acid is dependent on the chain length and the number of double bonds in the molecule, edible fats that are mixes of different fatty acids have comparable energy density [4]. Thus, the beneficial activity of PUFA-rich oils on CVD and other obesity-related disorders is believed to be associated with specific effects, including those on the blood lipid profile, blood pressure, adipocyte hormones, the inflammatory response and endothelial function along with many others that are both known and as yet undefined [4, 5].

In the scientific community, there is a tendency towards searching for alternative plant oils with an interesting fatty acid profile, biologically active components and potential health-promoting properties. One of these oils is blackcurrant seed oil, which contains considerable amounts of essential fatty acids. Apart from linoleic and *α*-linolenic acids, blackcurrant seed oil contains a small amount of stearidonic acid and is exceptionally abundant in γ-linolenic acid (up to 20 % of total fatty acids), both of which are uncommon components of plant oils [6, 7]. In the body, γ-linolenic acid is a precursor for the production of eicosanoids and can be synthesised to some limited extent from *α*-linoleic acid [8]. Furthermore, in addition to an interesting fatty acid profile, blackcurrant seed oil has also been reported to be a good source of tocopherols and sterols (1.1 g and 6.5 g/100 g of oil, respectively) [7]. Previous studies have demonstrated several biological activities of this oil, of which immunomodulating and anti-inflammatory are the most documented [8, 9]. Some clinical trials on the application of blackcurrant seed oil for the prevention of hypertension and atopic dermatitis have also been performed [10, 11].

In the present study, it was hypothesised that given the high nutritional value of blackcurrant seed oil and the potential biological activity of its components, the oil can beneficially modulate metabolic disorders related to obesity and its complications. A nutritional evaluation of blackcurrant seed oil and its effect on liver functions, inflammation markers and blood lipids were performed in rats fed a basal or obesogenic diet. Blackcurrant seed oil was added to the diets in place of rapeseed oil, which is an example of a common edible oil with well-established nutritional quality. Furthermore, the obesogenic diet contained a decreased level of complex carbohydrates and fibre and was high in fat, all of which can affect the gut microbiota [12, 13]; thus, the study also aimed to investigate its potentially adverse effects on the distal intestine.

## Materials and methods

### Dietary fats and their analysis

Refined low erucic acid rapeseed oil (ZT Kruszwica SA, Kruszwica, Poland) and pork lard (Animex Foods Ltd, Ostróda, Poland) were purchased in a local supermarket. The fatty acid profile of rapeseed oil was as follows: C16:0 (4.6 %), C18:0 (0.5 %), C18:1 n-9 (63.5 %), C18:2 n-6 (20.1 %), C18:3 n-3 (9.0 %) and C20:0 (2.2 %). The fatty acid profile of lard was as follows: C14:0 (1.6 %), C16:0 (29.3 %), C18:0 (0.5 %), C18:1 n-9 (58.1 %), C18:2 n-6 (8.9 %), C18:3 n-3 (0.6 %) and C20:0 (0.9 %). Refined blackcurrant seed oil of English origin was purchased from Greenaction (Kielce, Poland). The fatty acid proportions of the blackcurrant seed oil were within the ranges reported by Traifler et al. [6] and are given in Table 1.

**Table 1:** Fatty acid profile of blackcurrant seed oil (%)

C16:0	5.93 ± 0.76
C18:0	0.659 ± 0.004
C18:1 n-9	13.9 ± 0.1
C18:2 n-6	45.4 ± 0.2
C18:3 n-3	13.4 ± 0.0
C18:3 n-6	16.2 ± 0.2
C18:4 n-3	3.06 ± 0.03
C20:0	1.21 ± 0.44
C22:0	0.237 ± 0.028

The results are presented as the mean ± SD (*n* = 3)

The fatty acid profile of the fats was analysed after conversion of the fatty acids into methyl esters. An oil sample, together with pentadecanoic acid as an internal standard (0.1 μg/μL of oil), was dissolved in a solution composed of methanol/chloroform/sulphuric acid at a volumetric ratio of 100:100:1 and then heated at 100 °C for 2 h. Afterwards, an analysis was performed using a gas chromatograph (Clarus 600, Perkin Elmer, MA, USA) with a flame ionisation detector and a Supelcowax 10 column (30 m × 0.32 mm × 0.25 µm). The column, injector and flame ionisation detector temperatures were 190, 250 and 250 °C, respectively, and the flow rate of helium, used as a carrier gas, was 1.6 mL/min. Fatty acid methyl esters were identified using Totalchrom software.

### Animals and diets

The experiment was conducted on 32 male Wistar rats randomly assigned to one of four groups of eight animals each. The initial body weight was comparable among groups and equalled 128 ± 6 g on average. For 8 weeks, the rats were subjected to the following dietary treatments: groups B-RO and B-BO were fed a basal, semi-purified rodent diet [14] with rapeseed oil or blackcurrant seed oil as the sole source of fat, respectively (7 % of the diet), and groups O-RO and O-BO were fed an obesogenic diet containing rapeseed oil or blackcurrant seed oil, respectively (7 % of the diet). The obesogenic diet was a modification of the basal diet and contained lard and cholesterol (14 and 0.5 %, respectively, of the diet), which were added in place of a proportion of corn starch (Balviten Ltd, Katowice, Poland) and *α*-cellulose (Sigma-Aldrich). Details about the proportional composition of each group-specific diet are presented in Table 2. The animals were kept individually in cages under a 12-h light/dark cycle, a controlled temperature of 21–22 °C and intensive room ventilation (15 times per hour) and had free access to water and their respective diets. The diets were freshly prepared at weekly intervals and stored in hermetic containers at −20 °C. The use of animals complied with European guidelines for the care and use of laboratory animals, and the animal protocol employed in this study was approved by the Local Institutional Animal Care and Use Committee (Olsztyn, Poland).

**Table 2:** Composition of the group-specific diets

Ingredient (%)	Group			
	B-RO	B-BO	O-RO	O-BO
Casein	20	20	20	20
dl-Methionine	0.3	0.3	0.3	0.3
Lard^a^	–	–	14	14
Rapeseed oil (Canola)^b^	7	–	7	–
Blackcurrant seed oil^c^	–	7	–	7
Cholesterol	–	–	0.5	0.5
*α*-Cellulose	5	5	2	2
Sucrose	10	10	10	10
Corn starch	53.0	53.0	41.5	41.5
Mineral mix^d^	3.5	3.5	3.5	3.5
Vitamin mix^d^	1	1	1	1
Choline chloride	0.2	0.2	0.2	0.2
Calculated total fat content (%):	7.3	7.3	21.3	21.3
SFAs	0.51	0.56	5.03	5.08
MUFAs	4.45	0.97	12.58	9.11
PUFAs	2.04	5.46	3.37	6.79
n-6	1.41	4.31	2.65	5.56
n-3	0.63	1.15	0.71	1.24
Unidentified^e^	0.34	0.34	0.33	0.33
Energy density (kcal/g)	3.64	3.64	4.50	4.50

*SFAs* saturated fatty acids, *MUFAs* monounsaturated fatty acids, *PUFAs* polyunsaturated fatty acids

^a^Fatty acid profile: C14:0 (1.6 %), C16:0 (29.3 %), C18:0 (0.5 %), C18:1 n-9 (58.1 %), C18:2 n-6 (8.9 %), C18:3 n-3 (0.6 %) and C20:0 (0.9 %)

^b^Fatty acid profile: C16:0 (4.6 %), C18:0 (0.5 %), C18:1 n-9 (63.5 %), C18:2 n-6 (20.1 %), C18:3 n-3 (9.0 %) and C20:0 (2.2 %)

^c^Fatty acid profile in Table 1

^d^Recommended for the AIN-93G diet [14]

^e^From casein and corn starch preparations

### Sample collection and analysis

Upon termination of the experiment, the rats were weighed and anesthetised with sodium pentobarbital (50 mg/kg body weight). After a laparotomy, blood samples were collected from the caudal vein and stored in tubes containing ethylenediaminetetraacetic acid, and the gut segments (caecum and colon) and liver were removed and weighed.

The blood was centrifuged for 15 min at 380×*g*, and the obtained plasma was then stored at −20 °C until analysis. The plasma concentration of TGs, the total cholesterol (TC) and its HDL fraction (HDL-C) and the plasma activity of aspartate transaminase (AST) and alanine transaminase (ALT) were estimated using reagents from Alpha Diagnostics Ltd (Warsaw, Poland). Based on the plasma lipid profile, the atherogenic index was calculated as previously described, using the following formula: log(TGs (mmol/L)/HDL-C (mmol/L)) [15]. The plasma C-reactive protein (CRP) concentration was determined using a validated rat enzyme immunoassay kit (hs-CRP; Cusabio, Wuhan, China).

Samples of the caecal and colonic digesta were collected, fractions of which were stored at −70 °C. In the fresh caecal and colonic digesta, the pH was measured using a microelectrode and a pH/ION meter (model 301; Hanna Instruments, Vila do Conde, Portugal). The concentration of short-chain fatty acids (SCFAs) was measured in the caecal digesta using gas chromatography (Shimadzu GC-2010, Kyoto, Japan). Each sample (0.2 g) was mixed with 0.2 mL of formic acid, diluted with deionised water and centrifuged at 9,503×*g* for 10 min. The supernatant was loaded onto a capillary column (SGE BP21, 30 m × 0.53 mm; SGE Europe Ltd, MK, UK) using an on-column injector. The initial oven temperature was 85 °C and was increased to 180 °C at a rate of 8 °C/min and held there for 3 min. The temperatures of the flame ionisation detector and the injection port were 180 and 85 °C, respectively. The activity of the microbial enzymes (*α*- and *β*-glucosidase, *α*-and *β*-galactosidase) in the caecal and colonic digesta was measured spectrophotometrically using the rate of *p*- or *o*-nitrophenol release from their nitrophenyl-glycosides (Sigma-Aldrich) and expressed as µmol of product formed per h per g of digesta. The reaction mixture contained 0.3 mL of substrate solution (5 mM) and 0.2 mL of a 1:10 (v/v) dilution of the caecal or colon sample in 100 mM phosphate buffer (pH 7.0) after centrifugation at 9,503×*g* for 15 min. Incubation was performed at 37 °C, and *p*- and *o*-nitrophenol were quantified at 400 and 420 nm, respectively, after the addition of 2.5 mL of cold 0.25 M sodium carbonate.

The liver fat and lean mass were analysed shortly after dissection using time-domain nuclear magnetic resonance (Minispec LF 90II, Bruker, Karlsruhe, Germany). After storage of the liver at −20 °C, the glutathione (GSH) and glutathione disulphide (GSSG) levels in the liver tissue were determined spectrophotometrically using the method of Rahman et al. [16].

### Statistical analysis

The data are expressed as the mean and the pooled standard error of the mean (SEM). STATISTICA software, version 8.0 (StatSoft Corp., Krakow, Poland), was utilised to determine whether variables differed among the treatment groups. The effects of the type of diet (basal or obesogenic, *D*) and the type of dietary oil (rapeseed or blackcurrant seed, *O*) and the interaction between these investigated factors (*D* × *O*) were assessed by two-way analysis of variance (ANOVA). If the ANOVA revealed a significant interaction (*P* ≤ 0.05), the differences between the individual groups were then assessed with Duncan’s multiple range post hoc test at *P* ≤ 0.05.

## Results

After the 8-week period, for the obesogenic feeding regimen, the calorie intake (CI), the body weight (BW) and the body weight gain (BWG) were increased (*P* < 0.01, Table 3). Moreover, the obesogenic diet increased the relative liver mass and the liver’s fat proportion (*P* < 0.001 and *P* < 0.01), whereas the liver’s lean proportion was similar to the basal diet (*P* > 0.05). Compared with dietary rapeseed oil, dietary blackcurrant seed oil decreased the liver’s fat proportion (*P* < 0.05) but did not change the relative liver mass nor the liver’s lean proportion (*P* = 0.07). Moreover, the liver GSH/GSSG ratio were lowered by the obesogenic feeding regimen (*P* < 0.01).

**Table 3:** Animal growth and liver fat, lean and glutathione content in rats after 8 weeks of feeding

	CI (kcal/day)	BW (g)	BWG (g)	Liver			
				Mass^a^	Fat (%)	Lean (%)	GSH/GSSG^b^
Group							
B-RO	60.7	344	216	3.60	26.1	58.9	7.74
B-BO	62.3	356	228	3.42	25.1	59.0	6.74
O-RO	66.8	372	245	4.60	49.5	47.5	2.03
O-BO	67.7	380	252	4.26	40.1	53.8	1.85
Pooled SEM	0.94	5.1	5.2	0.100	2.22	1.40	0.748
Diet (*D*)							
Basal	61.5	350	222	3.51	25.6	59.0	7.24
Obesogenic	67.3	376	249	4.43	44.8	50.6	1.94
*P* value	0.001	0.007	0.005	0.000	0.002	0.146	0.009
Oil (*O*)							
Rapeseed	63.8	358	231	4.10	37.8	53.2	4.88
Blackcurrant seed	65.0	368	240	3.84	32.6	56.4	4.30
*P* value	0.061	0.072	0.054	0.284	0.041	0.065	0.518
Interaction (*D* × *O*)							
*P* value	0.102	0.204	0.184	0.800	0.148	0.236	0.276

Groups B-RO and B-BO were fed a basal diet containing rapeseed oil or blackcurrant seed oil, respectively, as the source of fat. Groups O-RO and O-BO were fed an obesogenic diet containing rapeseed oil or blackcurrant seed oil, respectively

*CI* calorie intake, *BW* body weight, *BWG* body weight gain, *GSH/GSSG* glutathione to glutathione disulphide ratio

^a^g/100 g body weight

^b^µmol/g tissue

**Fig. 1:**
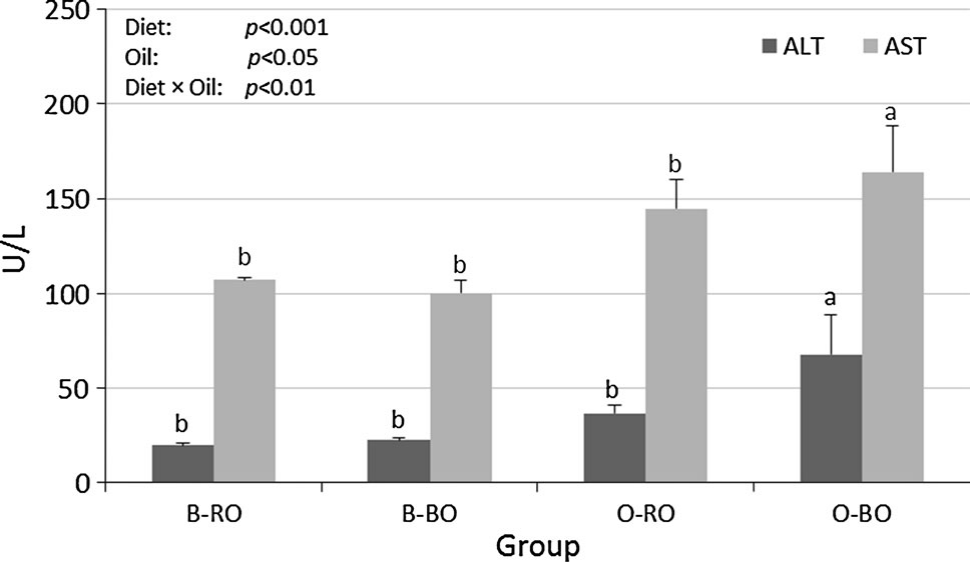
The plasma activity of alanine transaminase (ALT) and aspartate transaminase (AST) in rats. Groups B-RO and B-BO were fed a basal diet containing rapeseed oil or blackcurrant seed oil, respectively, as the source of fat. Groups O-RO and O-SO were fed an obesogenic diet containing rapeseed oil or blackcurrant seed oil, respectively. The mean values with unlike letters of the same colour (*a, b*) were significantly different in Duncan’s post hoc test (*P* < 0.05)

The plasma transaminase activities are shown in Fig. 1. Both the ALT and AST activity were affected by the type of diet (*P* < 0.001) and the type of oil (*P* < 0.01 and *P* < 0.05, respectively) and an interaction effect between these dietary factors was also observed (*D* × *O*, *P* < 0.01). Hence, the ALT and AST activity were higher in group O-BO than in the other groups, as established by the Duncan’s post hoc test (*P* ≤ 0.05).

The relative caecal and colon tissue and digesta mass and the pH value of the caecal and colon digesta were comparable among all the groups (*P* > 0.05, Table 4). The butyrate and total SCFA concentrations in the caecal digesta were affected by the obesogenic feeding regimen (*P* < 0.01 and *P* < 0.05, respectively). The activity of microbial enzymes in the hindgut digesta is shown in Table 5. In the caecal digesta, the *α*-and *β*-glucosidase and the *α*-galactosidase activity decreased due to the obesogenic diet (*P* < 0.01), and for the *α*-glucosidase activity, an interaction effect between the experimental factors was also observed (*D* × *O*, *P* < 0.01). The caecal *α*-glucosidase activity was higher in group B-BO than in the other groups (*P* ≤ 0.05). In the colon digesta, the obesogenic feeding regimen decreased the *β*-glucosidase and *α*-galactosidase activity (*P* < 0.001 and *P* < 0.01, respectively), whereas blackcurrant seed oil increased the *α*-and *β*-glucosidase and the *β*-galactosidase activity (*P* < 0.05 and *P* < 0.001 and *P* < 0.05, respectively). When examining the *β*-glucosidase activity, an interaction effect between the experimental factors was also observed (*D* × *O*, *P* < 0.01); thus, the activity was higher in group B-BO than in the other groups (*P* < 0.05).

**Table 4:** Basic indices and short-chain fatty acids (SCFAs) in the hindgut digesta of rats

	Caecum			Caecal SCFA concentration (μmol/g)				Colon		
	Tissue Mass^a^	Digesta mass^b^	pH	C2	C3	C4	Total	Tissue Mass^a^	Digesta mass^b^	pH
Group										
B-RO	0.164	3.55	7.19	120.0	20.4	26.9	176	0.268	1.042	7.13
B-BO	0.147	3.60	7.24	112.4	21.4	25.3	169	0.293	0.863	7.15
O-RO	0.146	3.15	7.31	98.9	20.8	11.0	138	0.242	0.830	7.35
O-BO	0.153	2.86	7.24	93.5	21.4	11.2	135	0.223	0.794	7.40
Pooled SEM	0.004	0.150	0.039	3.28	0.69	1.74	5.1	0.009	0.079	0.063
Diet (*D*)										
Basal	0.156	3.57	7.22	116.4	20.9	26.2	173	0.280	0.952	7.14
Obesogenic	0.149	3.01	7.28	96.2	21.1	11.1	136	0.232	0.812	7.38
*P* value	0.526	0.156	0.861	0.063	0.683	0.004	0.025	0.094	0.744	0.174
Oil (*O*)										
Rapeseed	0.155	3.35	7.25	109.4	20.6	19.0	157	0.255	0.936	7.24
Blackcurrant seed	0.150	3.23	7.24	102.3	21.4	17.8	151	0.258	0.828	7.29
*P* value	0.219	0.415	0.361	0.767	0.117	0.542	0.565	0.314	0.946	0.927
Interaction (*D* × *O*)										
*P* value	0.444	0.723	0.540	0.817	0.904	0.695	0.919	0.453	0.817	0.593

Groups B-RO and B-BO were fed a basal diet containing rapeseed oil or blackcurrant seed oil, respectively, as the source of fat. Groups O-RO and O-BO were fed an obesogenic diet containing rapeseed oil or blackcurrant seed oil, respectively. C2, acetate; C3, propionate; C4, butyrate

^a^g/100 g body weight

^b^g/g caecal or colon tissue

**Table 5:** Activity of microbial enzymes in the hindgut digesta of rats (μmol/h/g)

	Caecum				Colon			
	*α*-glu	*β*-glu	*α*-gal	*β*-gal	*α*-glu	*β*-glu	*α*-gal	*β*-gal
Group								
B-RO	19.8^b^	5.60	15.6	49.7	23.0	5.50^b^	20.2	66.0
B-BO	26.2^a^	6.99	22.9	54.1	32.4	11.3^a^	31.4	83.8
O-RO	19.1^b^	1.64	11.4	44.5	25.1	2.14^b^	14.7	67.0
O-BO	17.1^b^	2.21	12.9	44.4	27.3	5.02^b^	16.7	75.6
Pooled SEM	0.80	0.477	0.97	1.97	1.05	0.690	1.53	2.72
Diet (*D*)								
Basal	23.0	6.29	19.2	51.9	27.7	8.41	25.8	74.9
Obesogenic	18.1	1.93	12.2	44.5	26.2	3.58	15.7	71.3
*P* value	0.010	0.000	0.004	0.112	0.850	0.000	0.010	0.469
Oil (*O*)								
Rapeseed	19.4	3.62	13.5	47.1	24.1	3.82	17.4	66.5
Blackcurrant seed	21.7	4.60	17.9	49.2	29.9	8.17	24.0	79.7
*P* value	0.183	0.086	0.088	0.308	0.027	0.000	0.096	0.023
Interaction (*D* × *O*)								
*P* value	0.002	0.118	0.067	0.399	0.323	0.005	0.117	0.898

Groups B-RO and B-BO were fed a basal diet containing rapeseed oil or blackcurrant seed oil, respectively, as the source of fat. Groups O-RO and O-BO were fed an obesogenic diet containing rapeseed oil or blackcurrant seed oil, respectively

The mean values within a column with unlike superscript letters (a, b) were significantly different in Duncan’s post hoc test (*P* < 0.05)

*α*-glu, *α*-glucosidase; *β*-glu, *β*-glucosidase; *α*-gal, *α*-galactosidase; *β*-gal, *β*-galactosidase

The plasma lipids were considerably altered by the obesogenic diet (Table 6). The TG and HDL-C concentrations were decreased (*P* < 0.01), whereas the TC and non-HDL-C concentrations were increased (*P* < 0.01 and *P* < 0.001, respectively) by the obesogenic feeding regimen. When examining the HDL-C concentration, an interaction effect between the type of diet and the type of oil was observed; thus, the concentration was higher in group B-BO than in the other groups (*P* < 0.05). Moreover, dietary blackcurrant seed oil lowered the TG concentration and the atherogenic index (*P* < 0.01 and *P* < 0.05, Table 6).

**Table 6:** Lipid profile and atherogenic index of rat plasma

	TGs (mmol/L)	TC (mmol/L)	HDL-C (mmol/L)	Non-HDL-C (mmol/L)	Atherogenic index^a^
Group					
B-RO	2.198	1.863	1.315^b^	0.549	0.227
B-BO	1.243	2.114	1.513^a^	0.601	−0.095
O-RO	1.126	2.636	0.952^b^	1.684	0.055
O-BO	0.864	2.149	0.884^b^	1.266	−0.023
Pooled SEM	0.120	0.085	0.068	0.108	0.039
Diet (*D*)					
Basal	1.721	1.989	1.414	0.575	0.066
Obesogenic	0.995	2.393	0.918	1.475	0.019
*P* value	0.002	0.002	0.002	0.000	0.186
Oil (*O*)					
Rapeseed	1.662	2.250	1.134	1.116	0.141
Blackcurrant seed	1.053	2.132	1.199	0.933	−0.062
*P* value	0.007	0.123	0.258	0.299	0.016
Interaction (*D* × *O*)					
*P* value	0.320	0.286	0.049	0.854	0.360

Groups B-RO and B-BO were fed a basal diet containing rapeseed oil or blackcurrant seed oil, respectively, as the source of fat. Groups O-RO and O-SO were fed an obesogenic diet containing rapeseed oil or blackcurrant seed oil, respectively

The mean values within a column with unlike superscript letters (a, b) were significantly different in Duncan’s post hoc test (*P* < 0.05)

*HDL*-*C* HDL-cholesterol, Non-HDL-C, the difference between TC and HDL-C, *TC* total cholesterol, *TGs* triglycerides

^a^log(TG/HDL-C)

## Discussion

In this study, the energy density of the obesogenic diet was 0.86 kcal/g greater than that of the basal diet. This difference was caused by the lard addition in place of a proportion of corn starch and cellulose, which were the dietary carbohydrate and fibre sources, respectively. Although the diet intake (in grams) was significantly decreased by the obesogenic dietary regimen during the feeding period (data not shown), the overall difference in CI was sufficient to induce obesity by the end of the experiment. Furthermore, neither the CI nor the BWG was significantly affected by dietary blackcurrant seed oil (*P* = 0.061 and *P* = 0.054, respectively).

Recent studies have reported an aberrant gut microbiota in obese subjects which is connected with impaired microbial metabolic activities, especially the fermentation of non-digestible saccharides [17]. The most important end products of the fermentation process are SCFAs, primarily acetate, propionate and butyrate [18]. They are considered as indirect nutrients for the body that are readily absorbed from the distal intestine and have a role in the regulation of energy metabolism and many other metabolic features, like de novo lipogenesis [18, 19]. In the present study, the obesogenic dietary regimen lowered caecal butyrate and total SCFA concentration and altered microbial metabolic activity, as reflected by the decreased caecal and colonic glucosidase and galactosidase activities. The decrease in butyrate concentration is especially unfavourable because it is the preferred energetic substrate for colonocytes, which promotes a normal phenotype for these cells and protects against damage of their DNA [18, 20]. Notably, Jakobsdottir et al. [21] found that rats fed with a high-fat diet had significantly decreased levels of butyrate and increased levels of succinate both in the caecal digesta and serum. In human subjects, a high-fat and very low-carbohydrate diet was also able to decrease the faecal concentration of butyrate and total SCFAs [22]. A dietary component that increases SCFA production in the distal intestine, especially butyrate production, is resistant starch [20]. In this study, the amount of resistant starch was apparently on a lower level in the obesogenic diets, which contained less corn starch than the basal diets and that may explain the observed decrease in the butyrate concentration and *α*-glucosidase activity. The cellulose proportion was also decreased in the obesogenic diets; however, a previous study performed in our laboratory indicated that this dietary ingredient is largely inert to the caecal microbiota [23]. Thus, we speculate that the reduced microbial activity and SCFA production after the obesogenic dietary regimen was partly due to the decreased proportion of dietary fibre, including especially resistant starch. Notably, the study by De Wit et al. [24] suggests that a high dietary intake of fat can modulate the gut microbiota per se, likely due to its overflow into the distal intestine, and this effect seems to be dependent on the degree of unsaturation of fatty acids. In the present study, both the *α*- and *β*-glucosidase activities as well the *β*-galactosidase activity in the distal intestine were significantly elevated under the effect of dietary blackcurrant seed oil, whereas the obesogenic diet partly counteracted those effects. However, these results may also be a consequence of other components present in large quantities in blackcurrant seeds, like flavonoid glucosides and glucose and galactose polymers [25, 26], whose traces could have appeared in the oil after the extraction process.

In the present study, the obesogenic feeding regimen led to changes that can be considered typical for the development of fatty liver disease, including increased fat accumulation and deteriorated glutathione balance in the liver and elevated plasma transaminase activities [27]. In addition, the plasma C-reactive protein concentration was slightly but significantly increased after the obesogenic diet (data not shown). This finding indicates on a low-grade inflammation in the organism, which can be caused by endotoxins derived from intestinal bacteria and is involved in the development of fatty liver disease [28]. Cani et al. [29] showed that high-fat diet-induced changes in the gut microbiota led to the increase of intestinal permeability and thus to metabolic endotoxemia in mice. It is noteworthy that butyrate, whose caecal concentration in this study was much lower after the obesogenic diet than after the basal diet, seems to be a key bacterial metabolite that can maintain the intestinal barrier and reduce the progression of fatty liver disease [30, 31]. Furthermore, in the present study, dietary blackcurrant seed oil beneficially reduced the liver fat content, which is consistent with the findings of Traitler and Winter [32], who have demonstrated that feeding rats over a period of 3 months decreased the total lipids both in the liver and heart. However, both the plasma ALT and AST activities were detrimentally elevated in group O-BO, which appears to partly contradict the anti-inflammatory effects of blackcurrant seed oil widely suggested in the literature [8, 9]. Notably, both transaminase activities were not elevated when the blackcurrant seed oil was supplied with the basal diet. This phenomenon might suggest some problems with hepatic metabolism of the characteristic components of the oil, like γ-linolenic acid, when lipid metabolism is disturbed in the organism via an unbalanced diet. It is worth emphasising that both blackcurrant seed oil-containing diets had the same content of γ-linolenic acid (1.13 % of the diet, Table 2), and this acid was not present in the rapeseed oil-containing diets. To the best of our knowledge, specific tests of the liver function after ingestion of blackcurrant seed oil have not been performed thus far in either human or animal studies. However, there is a case study by Al-Khamees et al. [33] where the adverse effects of a γ-linolenic acid-rich borage oil have been described, including elevated transaminase levels. According to the available guidelines for preclinical studies, a two times or higher increase in plasma ALT activity should raise concerns on the hepatocellular injury [34]. In the present study, dietary blackcurrant seed oil increased the ALT activity 1.7 times on average, whereas the obesogenic dietary regimen increased the ALT activity 2.5 times on average. However, in three particular cases out of eight in group O-BO, the ALT activity was 2–5 times higher than the average activity noted in the O-RO group. This high variation can be partly explained by the individual response of rats to the diet as it was the case for mice chronically fed a high-fat diet [35].

In this study, the plasma lipid profile of rats was completely disturbed by the obesogenic diet. This was the result of the high dietary lard content, which significantly increased the content of SFAs and monounsaturated fatty acids (MUFAs), and the dietary presence of cholesterol (Table 2). The addition of blackcurrant seed oil to the basal and obesogenic diet notably increased the content of PUFAs and decreased the content of MUFAs compared to the corresponding rapeseed oil-containing diets. However, the difference in MUFA contents was much less significant between the obesogenic diets due to MUFAs that came from dietary lard. Nevertheless, the addition of blackcurrant seed oil beneficially lowered the TG concentration and the liver fat content regardless of the diet type, which thus appears to be due to the higher PUFA contents in the blackcurrant seed oil-containing diets. The beneficial effects of dietary PUFAs on lipid metabolism are mainly attributed to their ability for the induction of fatty acid oxidation in the liver and skeletal muscles and simultaneous suppression of hepatic lipid synthesis [36]. According to Gillingham et al. [37], PUFA-rich diets have slightly higher or comparable TC- and LDL-C-lowering activities as those of MUFA-rich diets; however, the former are more efficient in TG reduction, whereas the latter preserve better HDL-C. This is not in agreement with the present study in which the HDL-C concentration was beneficially increased only in the B-BO group whose dietary content of MUFAs was the lowest among all groups. Moreover, the addition of blackcurrant seed oil to the obesogenic diet did not increase the HDL-C concentration. Apparently, the disturbances in lipid metabolism caused by the obesogenic diet were too considerable to be overcome by the tested oil. Nevertheless, the aforementioned beneficial effects of blackcurrant seed oil are reflected in the lowered atherogenic index of plasma, which is inversely correlated with the lipoprotein particle size, thus predicting atherogenicity in humans [14]. In the literature, few nutritional experiments are available on the lipid-lowering effects of blackcurrant seed oil. Spielmann et al. [38] showed in hypercholesterolaemic patients that dietary supplementation with blackcurrant seed oil causes a decrease in plasma TC and LDL-C and an increase in HDL-C compared with grape seed oil supplementation. In another study, dietary supplementation with blackcurrant seed oil lowered LDL-C in healthy females compared with fish oil supplementation [39].

## Conclusions

The obesogenic dietary regimen causes obesity, alterations in the hindgut microbial metabolism, fatty liver, liver injury and oxidative stress and dyslipidaemia. When compared with rapeseed oil, dietary blackcurrant seed oil ameliorates the lipid metabolism of rats by lowering liver fat accumulation and the plasma TG level and atherogenicity as well by increasing the plasma HDL-C concentration. However, the beneficial effects are restricted when blackcurrant seed oil is provided together with an unbalanced diet, and a risk of liver injury may occur.

## References

[1] Astrup A, Dyerberg J, Selleck M, Stender S (2008). Nutrition transition and its relationship to the development of obesity and related chronic diseases. Obes Rev.

[2] Haggar FA, Boushey RP (2009). Colorectal cancer epidemiology: incidence, mortality, survival, and risk factors. Clin Colon Rectal Surg.

[3] Astrup A, Dyerberg J, Elwood P, Hermansen K, Hu FB, Jakobsen MU, Kok FJ, Krauss RM, Lecerf JM, LeGrand P, Nestel P, Risérus U, Sanders T, Sinclair A, Stender S, Tholstrup T, Willett WC (2011). The role of reducing intakes of saturated fat in the prevention of cardiovascular disease: where does the evidence stand in 2010?. Am J Clin Nutr.

[4] Lunn J, Theobald HE (2006). The health effects of dietary unsaturated fatty acids. Nutr Bull.

[5] Kim J, Li Y, Watkins BA (2013). Fat to treat fat: emerging relationship between dietary PUFA, endocannabinoids, and obesity. Prostaglandins Other Lipid Mediat.

[6] Traifler H, Wille HJ, Studer A (1988). Fractionation of blackcurrant seed oil. J Am Oil Chem Soc.

[7] Bakowska-Barczak AM, Schieber A, Kolodziejczyk P (2009). Characterization of canadian black currant (*Ribes nigrum L.*) seed oils and residues. J Agric Food Chem.

[8] Kapoor R, Huang YS (2006). Gamma linolenic acid: an anti-inflammatory omega-6 fatty acid. Curr Pharm Biotechnol.

[9] Linnamaa P, Nieminen K, Koulu L, Tuomasjukka S, Kallio H, Yang B, Tahvonen R, Savolainen J (2013). Black currant seed oil supplementation of mothers enhances IFN-γ and suppresses IL-4 production in breast milk. Pediatr Allergy Immunol.

[10] Deferne JL, Leeds AR (1996). Resting blood pressure and cardiovascular reactivity to mental arithmetic in mild hypertensive males supplemented with blackcurrant seed oil. J Hum Hypertens.

[11] Linnamaa P, Savolainen J, Koulu L, Tuomasjukka S, Kallio H, Yang B, Vahlberg T, Tahvonen R (2010). Blackcurrant seed oil for prevention of atopic dermatitis in newborns: a randomized, double-blind, placebo-controlled trial. Clin Exp Allergy.

[12] Murphy EF, Cotter PD, Healy S, Marques TM, O’Sullivan O, Fouhy F, Clarke SF, O’Toole PW, Quigley EM, Stanton C, Ross PR, O’Doherty RM, Shanahan F (2010). Composition and energy harvesting capacity of the gut microbiota: relationship to diet, obesity and time in mouse models. Gut.

[13] Chassard C, Lacroix C (2013). Carbohydrates and the human gut microbiota. Curr Opin Clin Nutr Metab Care.

[14] Reeves PG (1997). Components of the AIN-93 diets as improvements in the AIN-76A diet. J Nutr.

[15] Dobiásová M, Frohlich J (2001). The plasma parameter log (TG/HDL-C) as an atherogenic index: correlation with lipoprotein particle size and esterification rate in apoB-lipoprotein-depleted plasma (FER(HDL)). Clin Biochem.

[16] Rahman I, Kode A, Biswas SK (2006). Assay for quantitative determination of glutathione and glutathione disulfide levels using enzymatic recycling method. Nat Protoc.

[17] Conterno L, Fava F, Viola R, Tuohy KM (2011). Obesity and the gut microbiota: does up-regulating colonic fermentation protect against obesity and metabolic disease?. Genes Nutr.

[18] Wong JM, de Souza R, Kendall CW, Emam A, Jenkins DJ (2006). Colonic health: fermentation and short chain fatty acids. J Clin Gastroenterol.

[19] Delzenne NM, Cani PD (2011). Interaction between obesity and the gut microbiota: relevance in nutrition. Annu Rev Nutr.

[20] Conlon MA, Kerr CA, McSweeney CS, Dunne RA, Shaw JM, Kang S, Bird AR, Morell MK, Lockett TJ, Molloy PL, Regina A, Toden S, Clarke JM, Topping DL (2012). Resistant starches protect against colonic DNA damage and alter microbiota and gene expression in rats fed a western diet. J Nutr.

[21] Jakobsdottir G, Xu J, Molin G, Ahrne´ S, Nyman M (2013). High-fat diet reduces the formation of butyrate, but increases succinate, inflammation, liver fat and cholesterol in rats, while dietary fibre counteracts these effects. PLoS One.

[22] Brinkworth GD, Noakes M, Clifton PM, Bird AR (2009). Comparative effects of very low-carbohydrate, high-fat and high-carbohydrate, low-fat weight-loss diets on bowel habit and faecal short-chain fatty acids and bacterial populations. Br J Nutr.

[23] Jurgoński A, Juśkiewicz J, Zduńczyk Z (2008). Comparative effects of different dietary levels of cellulose and fructooligosaccharides on fermentative processes in the caecum of rats. J Anim Feed Sci.

[24] De Wit N, Derrien M, Bosch-Vermeulen H, Oosterink E, Keshtkar S, Duval C, des Vogel-van den Bosch J, Kleerebezem M, Muller M, van der Meer R (2012). Saturated fat stimulates obesity and hepatic steatosis and affects gut microbiota composition by an enhanced overflow of dietary fat to the distal intestine. Am J Physiol Gastrointest Liver Physiol.

[25] Lu YR, Foo LY (2003). Polyphenolic constituents of blackcurrant seed residue. Food Chem.

[26] Lengsfeld C, Deters A, Faller G, Hensel A (2004). High molecular weight polysaccharides from black currant seeds inhibit adhesion of Helicobacter pylori to human gastric mucosa. Planta Med.

[27] Schwenger KJ, Allard JP (2014). Clinical approaches to non-alcoholic fatty liver disease. World J Gastroenterol.

[28] Aron-Wisnewsky J, Gaborit B, Dutour A, Clement K (2013). Gut microbiota and non-alcoholic fatty liver disease: new insights. Clin Microbiol Infect.

[29] Cani PD, Bibiloni R, Knauf C, Waget A, Neyrinck AM, Delzenne NM, Burcelin R (2008). Changes in gut microbiota control metabolic endotoxemia-induced inflammation in high-fat diet-induced obesity and diabetes in mice. Diabetes.

[30] Peng L, Li ZR, Green RS, Holzman IR, Lin J (2009). Butyrate enhances the intestinal barrier by facilitating tight junction assembly via activation of AMP-activated protein kinase in Caco-2 cell monolayers. J Nutr.

[31] Endo H, Niioka M, Kobayashi N, Tanaka M, Watanabe T (2013). Butyrate-producing probiotics reduce nonalcoholic fatty liver disease progression in rats: new insight into the probiotics for the gut-liver axis. PLoS One.

[32] Traitler H, Winter H (1986). Fatty acid patterns in organ lipids in response to dietary black currant seed oil rich in gamma-linolenic acid. Prog Lipid Res.

[33] Al-Khamees WA, Schwartz MD, Alrashdi S, Algren AD, Morgan BW (2011). Status epilepticus associated with borage oil ingestion. J Med Toxicol.

[34] Boone L, Meyer D, Cusick P, Ennulat D, Bolliger AP, Everds N, Meador V, Elliott G, Honor D, Bounous D, Jordan H (2005). Selection and interpretation of clinical pathology indicators of hepatic injury in preclinical studies. Vet Clin Pathol.

[35] Duval C, Thissen U, Keshtkar S, Accart B, Stienstra R, Boekschoten MV, Roskams T, Kersten S, Müller M (2010). Adipose tissue dysfunction signals progression of hepatic steatosis towards nonalcoholic steatohepatitis in C57BL/6 mice. Diabetes.

[36] Clarke SD (2001). Polyunsaturated fatty acid regulation of gene transcription: a molecular mechanism to improve the metabolic syndrome. J Nutr.

[37] Gillingham LG, Harris-Janz S, Jones PJ (2011). Dietary monounsaturated fatty acids are protective against metabolic syndrome and cardiovascular disease risk factors. Lipids.

[38] Spielmann D, Traitler H, Crozier G, Fleith M, Bracco U, Finot PA, Berger M, Holman RT (1989). Biochemical and bioclinical aspects of blackcurrant seed oil: 3–6 balanced oil. NATO ASI A Ser.

[39] Tahvonen RL, Schwab US, Linderborg KM, Mykkänen HM, Kallio HP (2005). Black currant seed oil and fish oil supplements differ in their effects on fatty acid profiles of plasma lipids, and concentrations of serum total and lipoprotein lipids, plasma glucose and insulin. J Nutr Biochem.

